# Microarray-Based Comparative Genomics: Genome Plasticity in *Mycobacterium bovis*

**DOI:** 10.1002/cfg.182

**Published:** 2002-08

**Authors:** Jackie Inwald, Jason Hinds, James Dale, Si Palmer, Philip Butcher, R. Glyn Hewinson, Stephen V. Gordon

**Affiliations:** 1 TB Research Group Veterinary Laboratories Agency Woodham Lane New Haw Addlestone, Surrey KT15 3NB UK; 2 Bacterial MicroArray Facility Department of Medical Microbiology St Georges Hospital Medical School Cranmer Terrace London SE17 ORE UK


**Comparative and Functional Genomics**
*Comp Funct Genom* 2002; **3**: 342–344.
Published online in Wiley InterScience (www.interscience.wiley.com). **DOI:** 10.1002/cfg.182

**Conference Review**

### Microarray-based comparative genomics: genome plasticity in *Mycobacterium bovis*†

Jackie Inwald,[1] Jason Hinds,[2] James Dale,[1] Si Palmer,[1] Philip Butcher,[2] R. Glyn Hewinson[1] and Stephen V. Gordon[1]*

[1] *TB Research Group, Veterinary Laboratories Agency, Woodham Lane, New Haw, Addlestone, Surrey KT15 3NB, UK*
[2] *Bacterial MicroArray Facility, Department of Medical Microbiology, St Georges Hospital Medical School, Cranmer Terrace, London SE17 ORE, UK*

**Correspondence to:*
*Stephen V. Gordon, TB Research Group, Veterinary Laboratories Agency, Woodham Lane, New Haw, Addlestone, Surrey KT15 3NB, UK.*
*E-mail: svgordon@gtnet.gov.uk*

*† Neither Her Majesty's Stationery Office or the Veterinary Laboratories Agency accept any responsibility for the accuracy of any recipe, formula or instruction published in this article.*

Received: 31 May 2002
Accepted: 7 June 2002

### Introduction

*Mycobacterium bovis* is the causative agent of bovine tuberculosis, a disease responsible for annual losses to global agriculture of $3 billion and with serious repercussions for public health and animal welfare. The UK program for the control of bovine tuberculosis involves regular testing of cattle with a crude preparation of mycobacterial antigens (tuberculin), followed by compulsory slaughter of positive reactors. However, in the last decade the number of herd breakdowns has been increasing across the UK, especially in the south-west, where prevalence has now risen to 3.5% of cattle herds. This has worrying implications for the control strategy, which currently costs ~£25 million/year.

A range of techniques exist for the genetic typing of *M. bovis* isolates. These include restriction fragment length polymorphism (RFLP) with probes such as the polymorphic glycine-rich sequences (PGRS), a minisatellite method (VNTR), and spacer-oligonucleotide typing (spoligotyping). The application of these techniques has allowed the integration of molecular and epidemiological data to aid in disease control. The current method of choice for isolates at the Veterinary Laboratories Agency (VLA) is spoligotyping, a rapid simple method based on a polymorphic region called the direct repeat (DR) locus [1]. This locus is composed of multiple 36bp DR copies that are interspersed by non-repetitive, unique short sequences called spacers. Isolates of *M. bovis* differ in the presence or absence of spacers and adjacent DRs, allowing a barcode to be generated for each molecular type (Figure 1).

At the VLA approximately 16 000 strains have been spoligotyped. Analysis of this data shows that

© Crown Copyright 2002. Recorded with the permission of the Controller of her Majesty's Stationery Office.

| Strain | Spacer | | | | | | | | | | | | | | | | | | | | | | | | | | | | | | | | | | | | | |
|---|---|---|---|---|---|---|---|---|---|---|---|---|---|---|---|---|---|---|---|---|---|---|---|---|---|---|---|---|---|---|---|---|---|---|---|---|---|---|
| 09 | ■ | ■ | | ■ | ■ | | ■ | | | | | | ■ | ■ | ■ | | ■ | ■ | ■ | ■ | ■ | ■ | ■ | ■ | ■ | ■ | ■ | ■ | ■ | ■ | ■ | ■ | ■ | ■ | ■ | ■ | ■ | ■ |
| 17 | ■ | ■ | | ■ | ■ | | ■ | | | | | | ■ | ■ | ■ | | ■ | ■ | ■ | ■ | ■ | ■ | ■ | ■ | ■ | ■ | ■ | ■ | ■ | ■ | ■ | ■ | ■ | ■ | ■ | ■ | ■ | ■ |
| 12 | ■ | ■ | | ■ | ■ | | ■ | | | | | | ■ | ■ | ■ | | ■ | ■ | ■ | ■ | ■ | ■ | ■ | ■ | ■ | ■ | ■ | ■ | ■ | ■ | ■ | ■ | ■ | ■ | ■ | ■ | ■ | ■ |
| 11 | ■ | ■ | | ■ | ■ | | ■ | | | | | | ■ | ■ | ■ | | ■ | ■ | ■ | ■ | ■ | ■ | ■ | ■ | ■ | ■ | ■ | ■ | ■ | ■ | ■ | ■ | ■ | ■ | ■ | ■ | ■ | ■ |
| 13 | ■ | ■ | | ■ | ■ | | ■ | | | | | | ■ | ■ | ■ | | ■ | ■ | ■ | ■ | ■ | ■ | ■ | ■ | ■ | ■ | ■ | ■ | ■ | ■ | ■ | ■ | ■ | ■ | ■ | ■ | ■ | ■ |
| 10 | ■ | ■ | | ■ | ■ | | ■ | | | | | | ■ | ■ | ■ | | ■ | ■ | ■ | ■ | ■ | ■ | ■ | ■ | ■ | ■ | ■ | ■ | ■ | ■ | ■ | ■ | ■ | ■ | ■ | ■ | ■ | ■ |
| 22 | ■ | ■ | | ■ | ■ | | ■ | | | | | | ■ | ■ | ■ | | ■ | ■ | ■ | ■ | ■ | ■ | ■ | ■ | ■ | ■ | ■ | ■ | ■ | ■ | ■ | ■ | ■ | ■ | ■ | ■ | ■ | ■ |
| 20 | ■ | ■ | | ■ | | | | | | | | | | | ■ | | ■ | ■ | ■ | ■ | ■ | ■ | ■ | ■ | ■ | ■ | ■ | ■ | ■ | ■ | ■ | ■ | ■ | ■ | ■ | ■ | ■ | ■ |
| 25 | ■ | ■ | | ■ | ■ | ■ | ■ | ■ | | ■ | | | ■ | ■ | ■ | | ■ | ■ | ■ | ■ | ■ | ■ | ■ | ■ | ■ | ■ | ■ | ■ | ■ | ■ | ■ | ■ | ■ | ■ | ■ | ■ | ■ | ■ |
| 35 | ■ | ■ | | | | ■ | ■ | ■ | | ■ | ■ | ■ | ■ | ■ | ■ | | ■ | ■ | ■ | ■ | ■ | ■ | ■ | ■ | ■ | ■ | ■ | ■ | ■ | ■ | ■ | ■ | ■ | ■ | ■ | ■ | ■ | ■ |

**Figure 1.** Spoligotype patterns of dominant GB *M. bovis* molecular types. The spacer pattern at the DR locus is shown as blocks (■) to indicate the presence of a spacer, while empty lanes show the absence of a spacer. The strain numbers in the extreme left-hand column follow the VLA numbering convention. Spacers 39–43 are not shown in the UK there are only 10 major spoligotypes. Furthermore, two of these spoligotypes, 09 and 17, represent over 65% of all isolates. Type 09 is dispersed throughout the world, while Type 17 appears unique to Great Britain (GB), suggesting recent clonal expansion. Indeed, the majority of GB isolates can be related back to the Type 09, simply on the basis of spoligotype pattern (Figure 1). This suggests that DR deletions are clonal, and that progenitor clones would be predicted to have more spacers.

Prior to the availability of the *M. bovis* genome sequence, comparative genomics of the *M. tuberculosis* complex was performed using hybridization-based methods with micro- and macroarrays. These experiments revealed 10 deletions from the genome of *M. bovis*, ranging in size from ~1 to 12.7 kb [3,6]. The deletions impacted on a range of metabolic functions and putative virulence factors, e.g. loss of the RD5 locus removed the genes for three phospholipase C enzymes from the genome, a known virulence factor in *Listeria* and *Clostridium* spp. [7]. However, a fourth phospholipase gene, *plcD*, is intact in *M. bovis* and may compensate for the loss of the other genes. The RD7 locus encompasses one of the *mce* operons, originally described by Riley and colleagues as a putative mycobacterial invasin [2]. The genome sequence revealed that there are in fact four *mce* operons in *M. tuberculosis*, encoding a family of 24 proteins [5]. It is therefore possible that loss of one *mce* operon may be compensated by the remaining loci.

Analysis of the presence or absence of these deletions across the *M. tuberculosis* complex allowed a phylogenetic tree to be generated showing the evolutionary relationships between the strains [4]. From this analysis it was clear that the genome of *M. bovis* had undergone the greatest number of deletions, and that gene loss had been a major force in shaping the genome. However, the role these deletions played in the evolution of the bacillus is unclear. While they may represent host-adaptive mutations, it is also possible that they represent the fixation of deleterious mutations, or the removal of genetic redundancy. It is also unclear whether this process of deletion is continuing in 'modern' *M. bovis*.

### Results

The aim of this project is to determine whether clones of *M. bovis*, clustered on the basis of molecular type, share phenotypic characteristics that may explain their relative success as pathogens.

DNA microarray technology allows the large-scale analysis of whole genomes for comparative genomics. Using this technology we can therefore rapidly screen the genomes of *M. bovis* strains for deletions, using an *M. tuberculosis* H37Rv array and exploiting the >99.9% sequence identity between the two bacilli. This study will concentrate on the 10 most prevalent GB spoligotypes, i.e. Types 09,17,12,11,13,22,25,35,20 and 10. Our initial analysis has focused on the variation between the two dominant types, 09 and 17. Previously we had used a range of lipid profiling techniques to identify an alteration in the cell wall lipid profiles between these strains. However, due to the fact that ~10% of the coding capacity of the genome is dedicated to lipid metabolism, it was not possible to determine the genetic basis for this phenotype. The array-based approach has, however, identified

© Crown Copyright 2002

a deletion of a cluster of genes involved in lipid metabolism from *M. bovis* type 17. Deletion of this locus may therefore be responsible for the altered lipid profile of this strain, and we are actively pursing this possibility.

A second approach that we are taking is to determine whether deleted genes are immunogenic. Our analysis suggests that some of the deletions that we have identified are linked to spoligotype, in that every member of the same spoligotype shows the same deletion. Therefore, if any of the proteins encoded on these deletions encoded immunogenic epitopes, we may be able to use this as a basis for immunotyping. Our initial work focused on one gene deleted from Type 17. We used overlapping peptides to represent the encoded protein, and then screened these peptides in pools against whole blood from *M. bovis*-infected animals. Interferon-gamma (IFN-$\gamma$) concentration was determined using the BOVIGAM™ ELISA kit (Biocore AH, Omaha, NE). One pool of peptides from this protein was recognized by the majority of Type 09 *M. bovis* infected animals, offering the possibility of developing a rapid immunotyping method that would circumvent the need for culture of *M. bovis* from infected animals.

### Conclusions

Microarray-based comparative genomics allows genomes to be rapidly scanned for deletion events. This is a powerful method when it is linked to molecular epidemiological data, allowing the identification of genetic polymorphisms that could help explain phenotypic traits. This knowledge will help in our understanding of the mechanisms of genome variation and ultimately the evolution of the bacillus.

### Acknowledgements

We acknowledge BμG@S (the Bacterial Microarray Group at St George's) and the Wellcome Trust and Department of Environmental Food and Rural Affairs (DEFRA) for funding their work.

### References

1. Aranaz A, Liebana E, Mateos A, *et al.* 1996. Spacer oligonucleotide typing of *Mycobacterium bovis* strains from cattle and other animals: a tool for studying epidemiology of tuberculosis. *J Clin Microbiol* **34**: 2734–2740.
2. Arruda S, Bomfim G, Knights R, Huima-Byron T, Riley LW. 1993. Cloning of an *M. tuberculosis* DNA fragment associated with entry and survival inside cells. *Science* **261**: 1454–1457.
3. Behr MA, Wilson MA, Gill WP, *et al.* 1999. Comparative genomics of BCG vaccines by whole-genome DNA microarray. *Science* **284**: 1520–1523.
4. Brosch R, Gordon SV, Marmiesse M, *et al.* 2002. A new evolutionary scenario for the *Mycobacterium tuberculosis* complex. *Proc Natl Acad Sci USA* **99**: 3684–3689.
5. Cole ST, Brosch R, Parkhill J, *et al.* 1998. Deciphering the biology of *Mycobacterium tuberculosis* from the complete genome sequence. *Nature* **393**: 537–544.
6. Gordon SV, Brosch R, Billault A, Garnier T, Eiglmeier K, Cole ST. 1999. Identification of variable regions in the genomes of tubercle bacilli using bacterial artificial chromosome arrays. *Mol Microbiol* **32**: 643–655.
7. Titball RW. 1993. Bacterial phospholipases C. *Microbiol Rev* **57**: 347–366.

© Crown Copyright 2002 
